# A quick battery charging curve prediction by artificial neural network

**DOI:** 10.1016/j.patter.2021.100338

**Published:** 2021-09-10

**Authors:** Md Sazzad Hosen

**Affiliations:** 1 Battery Innovation Center, MOBI Research Group, Vrije Universiteit Brussel, Pleinlaan 2, 1050 Brussels, Belgium

## Abstract

Battery health prognosis and monitoring require the information of the available battery capacity that Tian et al. (2021) proposes to acquire from a partial 10-min charging curve via a deep neural network.

## Main text

The widespread use of battery-powered vehicles is critical to reducing environmental impact. The societal and regulatory changes are trending the development of carbon-emissions-free transport systems where lithium-ion (Li-ion) battery technologies are dominating as the main energy storage system. However, concern like battery health is still one of the challenging aspects to be addressed to eradicate range anxiety. To predict and monitor the state of health (SoH) of a battery is identified usually by health indicators extracted from a constant current charge and/or discharge curves that require a break of operation. In a real-life application, it is highly unlikely to get a full-fledged charging curve due to the fact that batteries are charged fast following different charging strategies. Thus, partial charging is usually what is available from the battery management system (BMS) to be considered for the SoH determination. In the June 16, 2021, issue of *Joule*, Tian et al. have proposed a deep neural network (DNN) that can predict the full charging curve from a 10-min partial constant-current (CC) curve and showcased a high accuracy through transfer learning as well.^1^ The estimated full charging curve provides information on the maximum available capacity and can be utilized for battery-state estimations and lifetime prediction.^2^

**Figure 1:**
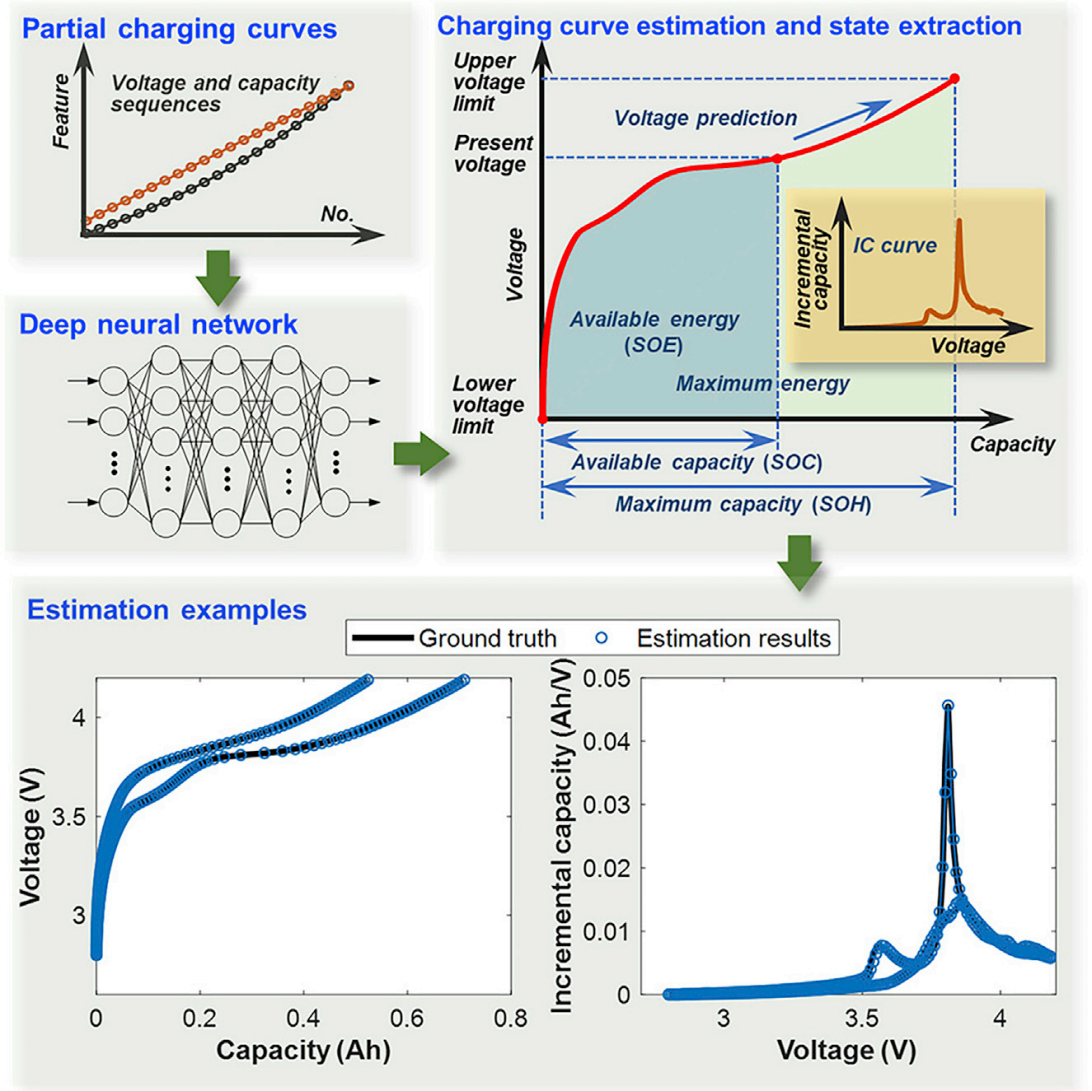
Overview of the quick charging curve prediction work by Tian et al.

It is a common practice to use partial segments of a full charging curve and/or discharged curve to extract health indicators parameterizing the estimation methods. For example, Feng et al. used a 15-min section from a full charging curve to estimate the SoH,^3^ Zheng et al. estimated battery capacity from charging curve sections,^4^ and the partial voltage-time data from the charge-discharge curve was employed by Richardson et al.^5^ However, a research gap is found when a quick charging scenario comes to play, and Tian et al. have demonstrated that it is good enough to predict the full curve with a DNN. Further, the research work trains and tests the developed model with several datasets showing the robustness and adaptability to an unknown set of inputs as well. Figure 1 displays the complete overview at a glance.

The use of complex DNNs is becoming popular in the battery field as it executes automatic featuring and optimal tuning and has flexible adaption to new problems compromising computational cost and complexity. Battery states can be derived quite precisely using deep networks.^6^^,^^7^ In the focused work,^1^ Adam’s algorithm is used to train the voltage sampling points to restore the entire charging curve by a developed DNN. The estimated curve is then used to predict the maximum capacity with around 4% maximum absolute estimation error and can also be used to construct an incremental capacity (IC) curve for battery-state estimation. The publicly available NASA dataset is used to train the network, while three more datasets are used to verify the performance showing capability of transfer learning.

The developed methodology paves a promising way of battery-state estimation that can be integrated into the BMS, enabling cloud simulation being in line with the real-life situation.^8^ However, the gathered knowledge still lacks variability, especially when so many battery technologies are available in the market. The trained DNN model would work for the different Li-ion chemistries in the same way or not that is yet to be seen. The different state of charge (SoC) regions may also yield prediction challenges, especially when temperature etc. dominate the open-circuit voltage. Further temperature and current rate variation may increase the robustness of the trained model. Moreover, a training dataset consisting of dynamic load variations draws more attention than lab-level constant current cycling results. Last but not the least, integration of the physics-informed degradation parameters is another viable prospect that can make the research work powerful and optimal.

Nevertheless, Tian et al. have already successfully showcased several of the challenging aspects of a full charging curve prediction. Overcoming the remaining challenges can only improve the existing work and results in a more robust and accurate simulation. The powerful tool can be implemented in the BMS to monitor the battery degradation receiving inputs from high-speed cloud simulation.
